# The blood urea nitrogen/creatinine (BUN/cre) ratio was U-shaped associated with all-cause mortality in general population

**DOI:** 10.1080/0886022X.2022.2030359

**Published:** 2022-02-15

**Authors:** Song Shen, Xudong Yan, Biao Xu

**Affiliations:** a Department of Cardiology, Affiliated Drum Tower Hospital of Nanjing University Medical School, Nanjing, China; b Department of Orthopedics, Nanjing Jiangning Hospital, Nanjing, China

**Keywords:** Blood urea nitrogen, creatinine, BUN/Cre ratio, all-cause mortality, NHANES

## Abstract

**Objectives:**

This study aimed to explore the relationship between the blood urea nitrogen/creatinine (BUN/Cre) ratio and all-cause or cause-specific mortality in the general population.

**Methods:**

Participants were enrolled from the National Health and Nutrition Examination Survey (NHANES) during 1999 to 2014. Baseline variables were acquired from questionnaires and examinations. Death status were ascertained from National Death Index records. Cox proportional hazards models with cubic spines were used to estimate hazard ratios (HRs) and 95% confidence interval (CI) of all-cause mortality, cardiovascular and cancer mortality.

**Results:**

A total of 42038 participants were enrolled in the study with a median 8.13 years of follow-up. Older people and women tend to have a higher BUN/Cre ratio. After multivariable adjustment, BUN/Cre ratio between 11.43 and 14.64 was associated with the lowest all-cause mortality compared with the participants with the lowest quartile (HR 0.83 [0.76, 0.91]; *p* < 0.001). The highest quartile of BUN/Cre ratio was associated with the lowest risk of cancer mortality (HR 0.64 [0.53, 0.78]; *p* < 0.001). Restricted cubic splines showed BUN/Cre was nonlinearly associated with all-cause mortality and linearly associated with cancer mortality.

**Conclusions:**

This study confirmed a U-shape relationship between BUN/Cre ratio and all-cause mortality in the general population.

## Introduction

The blood urea nitrogen/creatinine (BUN/Cre) ratio is a simple and widely used indicator for the assessment of renal function in clinic [1]. Blood urea nitrogen (BUN) is produced by the metabolism of protein and amino acids in the body, then hydrolyzed by the liver and excreted by the kidney with urine [2]. BUN reflects the physiological reactions of human protein intake and metabolism, liver and kidney function. Serum Cre is the product of muscle metabolism, and its value is related to the concentration of creatine in muscle [3]. In conclusion, the combination of BUN and Cre can reflect the muscle and protein metabolism.

Recently, it has been proposed as an effective prognostic factor for severe diseases, such as chronic heart failure [4], acute decompensated heart failure [5], gastrointestinal cancer [6], ischemic stroke [7] and septic shock [8]. Interestingly, higher levels of BUN or BUN/Cre were also associated with increased in-hospital mortality in patents with COVID-19 [9,10]. Many studies have reported the elevated BUN/Cre ratio was related to a poor prognosis and a higher mortality. However, little is known regarding whether elevated BUN/Cre ratio would increase the all-cause or cause-specific mortality in the general population. In this study, we aimed to explore the relationship between BUN/Cre ratio and all-cause or cause-specific mortality in the general population.

## Methods

### Study population

**Figure 1.**
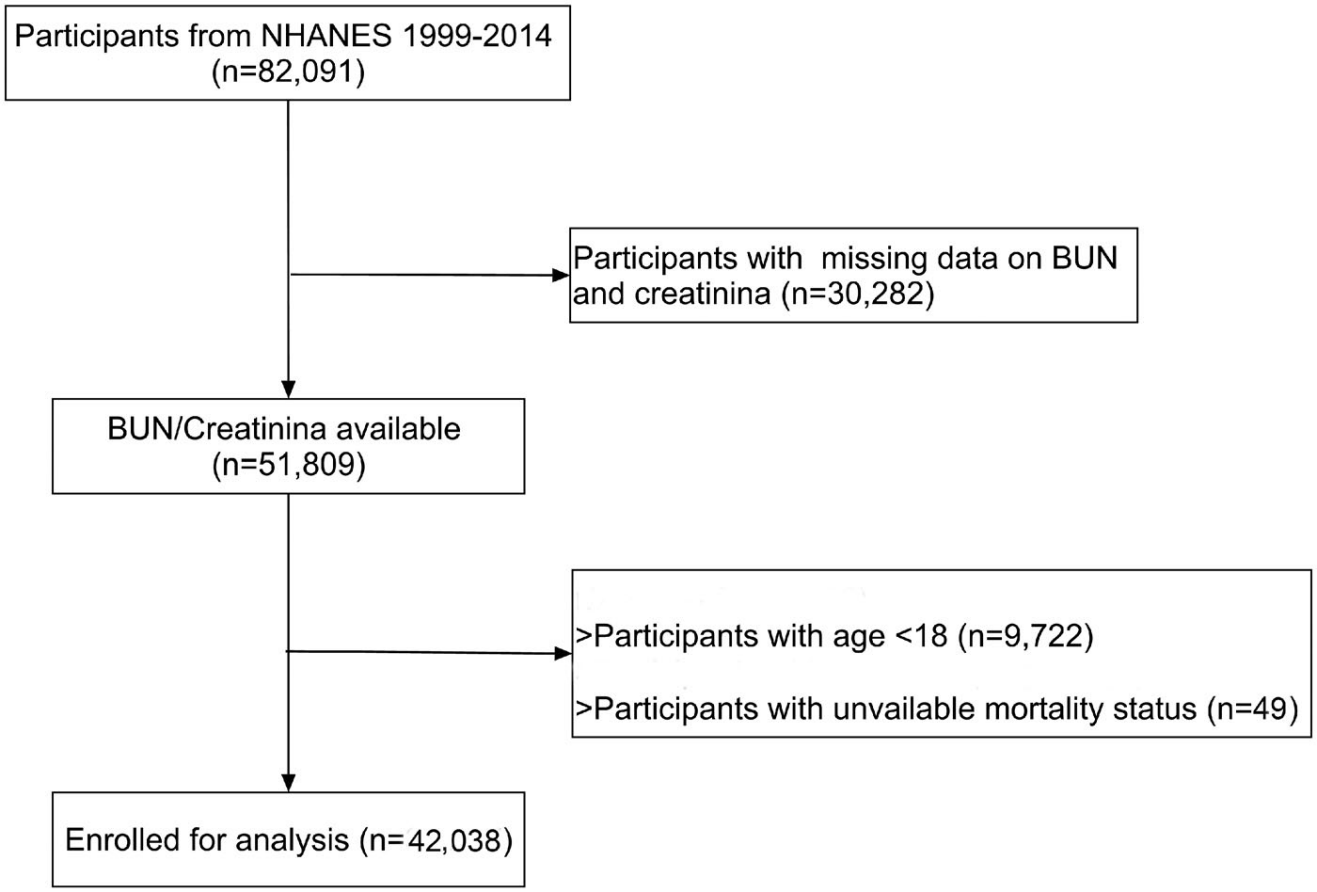
The flow chart of participant selection.

The National Health and Nutrition Examination Survey (NHANES) is a nationwide survey designed to assess the health and nutritional status of adults and children in the United States. The study used data from NHANES during 1999 to 2014. After excluding participants with missing data on BUN and Cre, age <18 years old and unavailable mortality status, 42038 participants were enrolled in the study (Figure 1). The study was approved by the institutional review board of Nanjing University (2020AE01065) and all participants provided written informed consent.

### Exposure and outcomes

The blood urea nitrogen was assessed using an enzymatic conductivity rate method. Serum Cre was measured using the kinetic Jaffe rate method. The main outcomes of the study were all-cause mortality, cardiovascular mortality and cancer-related mortality. Mortality status was obtained by linkage to the National Death Index by 31 December 2015. Cardiovascular disease was defined as ICD-10 codes I00-I09, I11, I13, or I20-I51. Malignant neoplasm was defined as ICD-10 codes C00-C97.

### Other clinical characteristics

The baseline characteristics of participants were acquired by questionnaires and examinations, including demographics (such as sex, age, race, educational level, and family poverty income ratio), lifestyle information (such as physical activity, drinking and smoking), medical history (hypertension and cardiovascular diseases) and mediations use (antihypertensive drugs, hypoglycemic drugs and lipid-lowering drugs). Race was classified as non-Hispanic white, non-Hispanic black, Mexican American, other Hispanic or others. Education level was categorized as less than high school, high school, equivalent and college or above. Family poverty income ratio (PIR) was stratified as <1, 1–3, and > 3. Smoking status were defined as current, past and never. Physical activity status was classified as vigorous, moderate and inactive. Hypertension was defined as a history of hypertension or blood pressure ≥140/90 mmHg or taking anti-hypertensive medications. The cardiovascular disease (CVD) was defined as self-reported congestive heart failure, coronary heart disease, angina pectoris, heart attack and stroke. The estimated glomerular filtration rate (eGFR) was calculated according to the Chronic Kidney Disease-Epidemiology Collaboration (CKD-EPI) equation. Multiple imputation (*n* = 5) using predictive mean matching (PMM) was performed for covariates with missing values, including education level, race, PIR, body mass index, smoker, drinker, physical activity, high blood pressure, diabetes mellites, cardiovascular diseases, antihypertensive drugs, hypoglycemic drugs, and lipid-lowering drugs.

### Statistical analysis

Continuous variables were described as the mean ± standard deviation and categorical variables were presented as numbers and proportions. Sample weight were used to estimate the representative distribution using the supplied masked variance pseudo-stratum and masked variance pseudo-primary sampling units. Differences between groups were explored by one-way analysis of variance (ANOVA), or chi-square tests. Univariate survival analysis was performed with Kaplan–Meier analysis and Log rank test. Multivariate Cox proportional hazards regression models were used to estimate hazard ratios (HRs) and 95% confidence interval (CI) for all-cause, cardiovascular, and cancer mortality. Model 1 was adjusted for age and gender. Model 2 was adjusted for age, gender, education level, race, PIR, body mass index, smoker, drinker, physical activity. Model 3 was adjusted for age, gender, education level, race, PIR, body mass index, smoker, drinker, physical activity, high blood pressure, diabetes mellites, cardiovascular diseases, antihypertensive drugs, hypoglycemic drugs, lipid-lowering drugs and eGFR. Restricted cubic spline with 3 knots (0.10, 0.50, and 0.90 respectively) based on the Model 3 was used to describe the nonlinear relationship between BUN/Cre ratio and mortality with Wald χ2 test. Subgroup analyses was used to explore the interactive effect of gender, age, race, education, PIR, and comorbidities. All statistical analyses were performed using R version 3.6, and *p* < 0.05 was considered as statistically significant.

## Results

The study included 42038 participants with a median 8.13 years of follow-up. Compared to participants with lower BUN/Cre ratio, population with higher BUN/Cre ratio were older, more females, more drinkers and had more percentage of hypertension, diabetes and cardiovascular diseases (Table 1).

**Table 1.** Characteristics of the study population.

Variable	BUN/Cr*e* < 11.43 (*n* = 10514)	BUN/Cr*e* < 14.64 (*n* = 10542)	BUN/Cr*e* < 18.57 (*n* = 10412)	BUN/Cr*e* ≥ 18.57 (*n* = 10570)	*p* Value
Male (%)	6050 (57.5)	5853 (55.5)	4870 (46.8)	3602 (34.1)	<0.001
Age, years	40.61 (17.46)	45.04 (18.75)	49.07 (19.40)	53.70 (19.44)	<0.001
Race (%)					<0.001
Non-Hispanic white	4165 (39.6)	4865 (46.1)	5150 (49.5)	5089 (48.1)	
Non-Hispanic black	3774 (35.9)	2406 (22.8)	1459 (14.0)	984 (9.3)	
Mexican American	1392 (13.2)	1789 (17.0)	2115 (20.3)	2792 (26.4)	
Others	1183 (11.3)	1482 (14.1)	1688 (16.2)	1705 (16.1)	
Education (%)					<0.001
Less than high school	2408 (25.8)	2441 (25.1)	2715 (27.7)	3466 (34.6)	
High school or equivalent	2318 (24.8)	2219 (22.8)	2236 (22.9)	2238 (22.4)	
College or above	4609 (49.4)	5052 (52.0)	4834 (49.4)	4308 (43.0)	
PIR (%)					<0.001
<1	2628 (26.7)	2069 (21.2)	1938 (20.3)	1936 (20.4)	
1 ∼ 3	3979 (40.4)	3975 (40.7)	3887 (40.7)	4115 (43.4)	
>3	3249 (33.0)	3711 (38.0)	3733 (39.1)	3428 (36.2)	
BMI, kg/m^2^	28.32 (6.77)	28.55 (6.64)	28.70 (6.66)	28.55 (6.58)	0.001
Drinking (%)	28.32 (6.77)	28.55 (6.64)	28.70 (6.66)	28.55 (6.58)	0.001
Smoking (%)					<0.001
Current	2537 (33.4)	1830 (24.6)	1405 (19.7)	1115 (15.5)	
Past	436 (5.7)	424 (5.7)	338 (4.7)	285 (4.0)	
Never	4621 (60.9)	5183 (69.7)	5406 (75.6)	5786 (80.5)	
Activity (%)					<0.001
Vigorous	2954 (45.1)	2749 (42.6)	2411 (40.2)	2017 (37.0)	
Moderate	2470 (37.7)	2558 (39.7)	2553 (42.6)	2510 (46.0)	
Inactive	1133 (17.3)	1144 (17.7)	1028 (17.2)	924 (17.0)	
Medical history (%)					
HBP	1637 (16.3)	1749 (17.3)	1836 (18.4)	2410 (23.9)	<0.001
DM	1043 (9.9)	1295 (12.3)	1608 (15.4)	2059 (19.5)	<0.001
CVD	612 (6.5)	761 (7.8)	916 (9.4)	1093 (10.9)	<0.001
Cancers	598 (5.9)	778 (7.4)	937 (9.0)	1134 (10.7)	<0.001
Antihypertensive drugs	2113 (77.8)	2541 (81.5)	2891 (85.3)	3620 (89.1)	<0.001
Hypoglycemic drugs	476 (45.0)	675 (51.3)	903 (56.1)	1224 (61.3)	<0.001
Lipid-lowering drugs	1060 (77.5)	1403 (79.7)	1632 (80.8)	2016 (82.2)	0.004
Systolic BP, mmHg	121.99 (17.76)	123.02 (19.33)	124.24 (20.12)	127.01 (22.12)	<0.001
Diastolic BP, mmHg	70.15 (13.61)	69.85 (13.41)	69.50 (13.43)	68.73 (14.38)	<0.001
HbA1c, %	5.50 (0.91)	5.58 (0.93)	5.67 (1.03)	5.79 (1.21)	<0.001
Cholesterol, mg/dL	70.15 (13.61)	69.85 (13.41)	69.50 (13.43)	68.73 (14.38)	<0.001
eGFR, ml/min per 1.73 m^2^	91.73 (26.13)	92.86 (26.63)	96.18 (31.12)	105.53 (44.57)	<0.001
BUN, mg/dL	8.86 (4.55)	11.92 (3.70)	14.19 (4.57)	17.79 (6.83)	<0.001
Cre, mg/dL	0.99 (0.76)	0.91 (0.28)	0.86 (0.27)	0.78 (0.28)	<0.001
BUN/Cre ratio	9.16 (1.80)	13.09 (0.90)	16.47 (1.09)	23.15 (4.60)	<0.001

Data are presented as mean (SD) or *n* (%). PIR: poverty income ratio; BMI: body mass index; HBP: high blood pressure; DM: diabetes mellites; CVD: cardiovascular diseases; eGFR: estimated glomerular filtration rate; BUN: blood urea nitrogen; Cre: creatinine.

**Figure 2.**
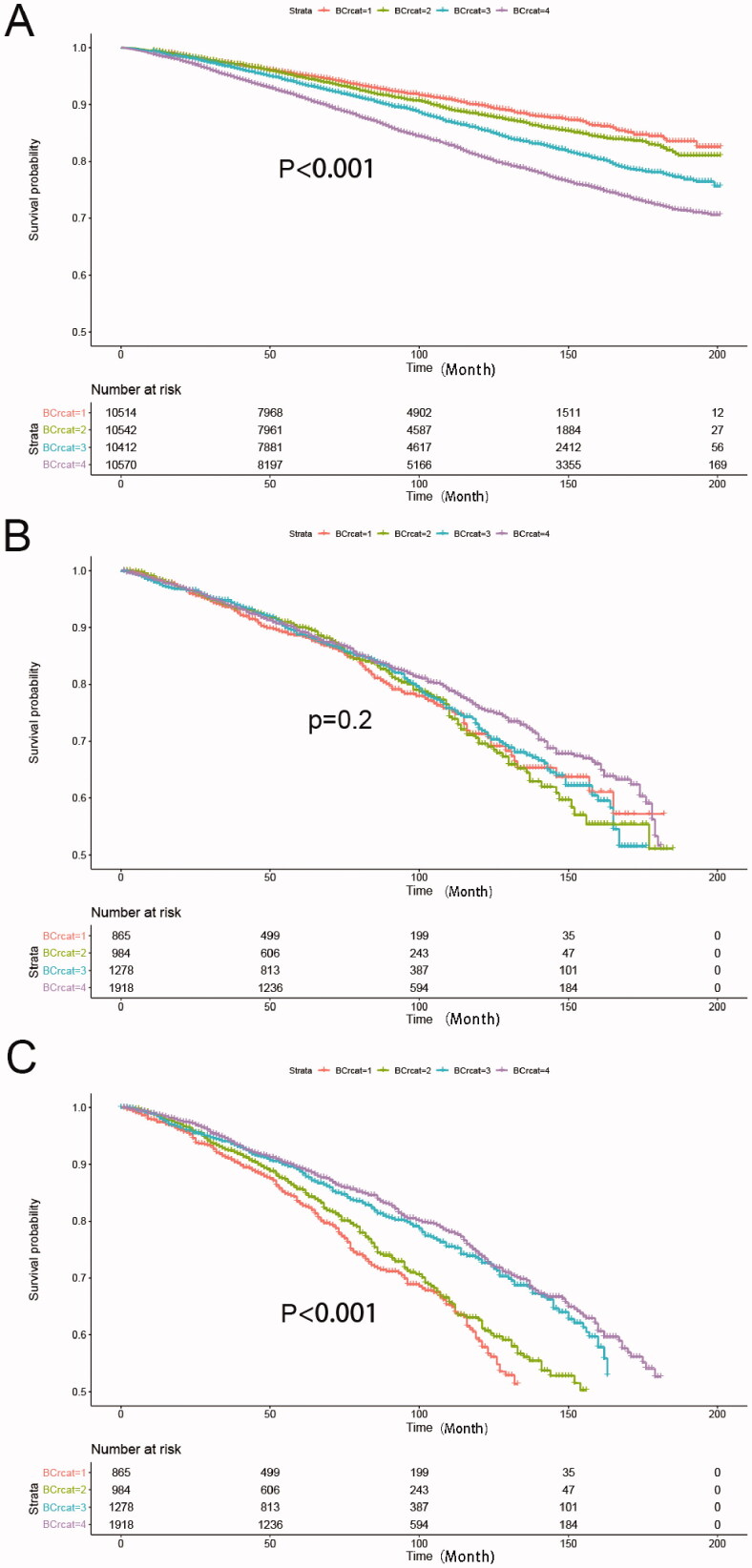
The Kaplan–Meier analysis between the BUN/Cre ratio quartiles and all-cause mortality (A), cardiovascular mortality (B) and cancer mortality (C). BCrcat represented BUN/Cre quartiles. The P values were calculated by the log-rank test.

As shown in Figure 2, Kaplan-Meier analysis suggested that lower BUN/Cre ratio was associated with a lower all-cause mortality in the whole population. After adjusting for demographics, lifestyles and medical history, we found that BUN/Cre ratio between 11.43 and 14.64 was associated with the lowest all-cause mortality compared with the lowest quartile (HR 0.83[0.76, 0.91]; *p*<0.001; Table 2). In addition, a negative correlation was observed between BUN/Cre ratio and cancer-related mortality. The highest quartile of BUN/Cre ratio was associated with the lowest risk of cancer mortality (HR 0.64 [0.53, 0.77]; *p* < 0.001). However, the relationship between BUN/Cre ratio and cardiovascular mortality disappeared after multivariable adjustment.

**Table 2.** Association of BUN/Cre ratio with all-cause and cause specific mortality.

All cause and cause specific mortality	Cases	*N*	Model 1 HR(95% CI)	Model 2 HR(95% CI)	Model 3 HR(95% CI)
All causes					
Q1	867	10514	Ref	Ref	Ref
Q2	986	10542	0.76 [0.70, 0.84]***	0.82 [0.75, 0.90]***	0.83 [0.76, 0.91]***
Q3	1281	10412	0.77 [0.70, 0.84]***	0.87 [0.79, 0.95]***	0.87 [0.80, 0.95]**
Q4	1923	10570	0.84 [0.77, 0.92]***	0.97 [0.89, 1.06]	1.03 [0.94, 1.12]
Continuous	5057	42038	1.00 [0.99, 1.00]	1.01 [1.00, 1.01]*	1.01 [1.01, 1.02]***
Cardiovascular					
Q1	139	865	Ref	Ref	
Q2	165	984	0.88 [0.70, 1.10]	0.82 [0.75, 0.90]***	0.87 [0.69, 1.09]
Q3	235	1278	0.81 [0.66, 1.01]	0.87 [0.79, 0.95]**	0.83 [0.66, 1.03]
Q4	335	1918	0.74 [0.60, 0.91]**	0.97 [0.89, 1.06]	0.82 [0.65, 1.02]
Continuous	874	5045	0.98 [0.97, 0.99]**	0.99 [0.98, 1.00]	0.99 [0.98, 1.01]
Malignant neoplasms					
Q1	220	705	Ref	Ref	Ref
Q2	231	744	0.86 [0.72, 1.04]	0.88 [0.73, 1.06]	0.88 [0.72, 1.06]
Q3	249	1016	0.63 [0.53, 0.76]***	0.67 [0.55, 0.81]***	0.66 [0.55, 0.80]***
Q4	359	1537	0.58 [0.49, 0.69]***	0.62 [0.52, 0.75]***	0.64 [0.53, 0.77]***
Continuous	757	4002	0.96 [0.95, 0.97]***	0.97 [0.96, 0.98]***	0.99 [0.98, 1.01] ***

Model 1 was adjusted for age and gender.

Model 2 was adjusted for age, gender, education level, race, poverty income ratio, body mass index, smoker, drinker, physical activity.

Model 3 was adjusted for age, gender, education level, race, poverty income ratio, body mass index, smoker, drinker, physical activity, high blood pressure, diabetes mellites, cardiovascular diseases, cancers, antihypertensive drugs, hypoglycemic drugs, lipid-lowering drugs, sBP, dBP, HbA1c, cholesterol and eGFR.

*p < 0.5, **p < 0.01, ***p < 0.001; HR, hazard ratio; CI, confidence interval.

**Figure 3.**
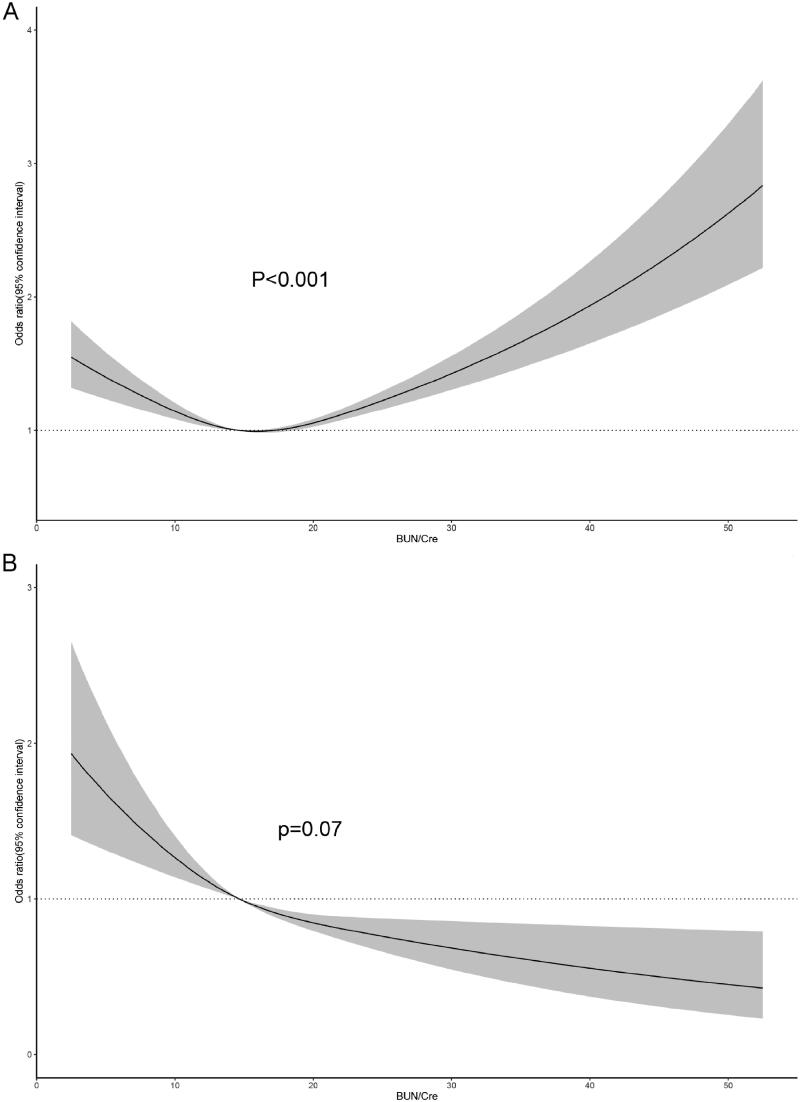
The restricted cubic regression between the BUN/Cre ratio with all-cause mortality (A), and cancer mortality (B) in Model 3. The P values were calculated by the Wald χ2 test.

To explore the nonlinear relationship between BUN/Cre ratio and all-cause mortality as well as cancer mortality, restricted cubic spines based on Cox regression models were performed (Figure 3). We found a nonlinear association existed in all-cause mortality **(**Figure 3(A)) while a linear relationship in cancer mortality (Figure 3(B)). Subgroup analysis (Table 3) showed that the association was consistent across groups except for the history of CVD. The BUN/Cre ratio was U-shaped associated with the risk of all-cause mortality in participants without CVD while increased the risk of mortality in participants with CVD.

**Table 3.** Subgroup analysis of association of BUN/Cre ratio with all-cause mortality.

	BUN/Cre	Q1	Q2	Q3	Q4	P for interaction
**Gender**						0.721
Female	16.6 ± 6.28	Ref	0.79 [0.67, 0.93]**	0.76 [0.65, 0.89]***	0.95 [0.82, 1.10]	
Male	14.3 ± 4.87	Ref	0.85 [0.76, 0.96]**	0.94 [0.84, 1.05]	1.05 [0.94, 1.18]	
**Age**						0.188
<60	14.6 ± 5.42	Ref	0.87 [0.74, 1.03]	0.77 [0.65, 0.92]**	0.82 [0.68, 0.99]*	
≥60	17.3 ± 6.02	Ref	0.80 [0.71, 0.89]***	0.86 [0.77, 0.96]**	1.05 [0.94, 1.17]	
**Race**						0.054
Non-Hispanic white	15.8 ± 5.56	Ref	0.77 [0.68, 0.88]***	0.81 [0.72, 0.92]**	0.94 [0.83, 1.06]	
Non-Hispanic black	12.8 ± 4.80	Ref	0.84 [0.70, 1.00]*	0.91 [0.75, 1.10]	1.38 [1.12, 1.69]**	
Mexican American	17.1 ± 6.28	Ref	0.95 [0.74, 1.22]	0.90 [0.72, 1.14]	1.01 [0.80, 1.26]	
Others	16.1 ± 5.57	Ref	0.70 [0.48, 1.02]	0.61 [0.42, 0.88]**	0.81 [0.57, 1.17]	
**Education**						0.534
Less than high school	16.3 ± 6.37	Ref	0.86 [0.75, 1.00]*	0.92 [0.80, 1.06]	1.11 [0.97, 1.27]	
High school or equivalent	15.2 ± 5.67	Ref	0.75 [0.62, 0.90]**	0.83 [0.69, 1.00]*	0.88 [0.74, 1.06]	
College or above	15.1 ± 5.34	Ref	0.86 [0.73, 1.01]	0.83 [0.71, 0.98]*	1.01 [0.86, 1.19]	
**PIR (%)**						0.053
<1	15.0 ± 5.96	Ref	0.91 [0.76, 1.10]	0.94 [0.79, 1.13]	1.01 [0.84, 1.22]	
1 ∼ 3	15.6 ± 5.86	Ref	0.80 [0.70, 0.90]***	0.86 [0.76, 0.97]*	1.05 [0.93, 1.19]	
>3	15.6 ± 5.47	Ref	0.88 [0.72, 1.07]	0.82 [0.67, 0.99]*	0.98 [0.81, 1.18]	
**HBP**						0.578
No	15.3 ± 5.60	Ref	0.84 [0.75, 0.95]**	0.89 [0.79, 1.00]*	1.03 [0.92, 1.16]	
Yes	16.3 ± 6.26	Ref	0.79 [0.69, 0.92]**	0.80 [0.69, 0.93]**	0.99 [0.86, 1.14]	
**DM**						0.393
No	15.2 ± 5.64	Ref	0.85 [0.76, 0.94]***	0.87 [0.78, 0.97]*	0.98 [0.88, 1.09]	
Yes	17.0 ± 6.16	Ref	0.82 [0.68, 0.99]*	0.88 [0.74, 1.05]	1.13 [0.95, 1.34]	
**CVD**						**0.001**
No	15.5 ± 5.72	Ref	0.83 [0.74, 0.92]***	0.87 [0.78, 0.96]**	0.93 [0.84, 1.04]	
Yes	16.7 ± 6.09	Ref	0.87 [0.72, 1.06]	0.93 [0.77, 1.12]	1.30 [1.08, 1.57]**	

## Discussion

Our study found a U-shape association between the BUN/Cre ratio and all-cause mortality, namely a lower BUN/Cre ratio and higher BUN/Cre ratio were all related to a higher all-cause mortality. Besides, a higher BUN/Cre ratio was linearly associated with the lower risk of cancer mortality.

BUN and Cre are the end products of nitrogen metabolism in human body, whose change reflect the renal function, nutritional and metabolic status. BUN/Cre ratio is one of the common indices used to separate pre-renal azotemia and acute tubular necrosis, with a threshold of 20 [11]. Previous studies have found that a higher BUN/Cre ratio was associated with the worse prognosis in HF [4,12], stroke [13], upper gastrointestinal bleeding [14], hemodialysis [15], and septic shock [8]. Recently, it was reported that BUN/Cre ratio was a predictor of disease severity and survival of COVIPD-19 patients [9,16]. In general population, BUN/Cre ratio was negatively associated with the incidence of stroke [17]. Yuya *et al* reported that the median BUN/Cre ratio in the general population was 15.0 (12.9–17.6) [12]. We have found a U-shape association between BUN/Cre ratio and all-cause mortality in general population. When BUN/Cre ratio was between 11.43 and 14.64, it was associated with the lowest risk of mortality. This range may represent an ideal protein intake and metabolism in human. Higher levels of BUN/Cre reflect a more active neurohormonal system, contributing to adverse prognosis [18]. While low BUN/Cre ratio levels may be related to inflammation, oxidative stress and endothelial dysfunction after acute renal injury [19].

We have also observed a negative relationship between BUN/Cre ratio and cancer mortality. A retrospective study reported that elevated Cre was related to increased mortality in patients with malignant fibroblastic and myofibroblast sarcomas [20] and liposarcoma [21]. However, studies on BUN/Cre ratio and cancer-related mortality are scarce and the mechanism deserves further exploration. The relationship between BUN/Cre ratio and cardiovascular-specific mortality disappeared after adjusting for multiple covariates. We hypothesized that BUN/Cre ratio predict the prognosis of heart failure by reflecting the change of volume load. As cardiovascular disease-related mortality is a relatively broad concept, further work should focus on the concrete cardiovascular disease.

Some limitations existed in our study. Firstly, the disease history was self-reported by participants, which may cause recall errors. Secondly, one-time measurement of BUN/Cre would vary by the participants’ condition and was not a long-term reflection.

## Conclusions

In conclusion, we reported a U-shape relationship between BUN/Cre ratio and all-cause mortality in general population. Conversely, BUN/Cre ratio was negatively correlated with cancer mortality. Further studies will be needed to determine whether interfering BUN/Cre ratio will improve the long-term prognosis in general population.

## Ethical approval

The study was approved by the institutional review board of Nanjing University (2020AE01065) and all participants provided written informed consent.

## Data Availability

The data supporting our results were available in NHANES.
